# Cross-sectoral sharing of CTX-M-producing *Escherichia coli*: a One Health analysis to understand dissemination modes

**DOI:** 10.1128/spectrum.03551-25

**Published:** 2026-02-19

**Authors:** Shuo Jiang, Hangshu Fang, You-Xiang Chan, Kin-Hung Chow, Pak-Leung Ho, Huiluo Cao

**Affiliations:** 1 Department of Microbiology, University of Hong Kong, Hong Kong SAR, People’s Republic of China; 2 Agricultural Genomics Institute at Shenzhen, Chinese Academy of Agricultural Sciences, Shenzhen, China; 3 Carol Yu Center for Infection, University of Hong Kong, Hong Kong SAR, People’s Republic of China; Tel Aviv Sourasky Medical Center, Tel Aviv, Israel

**Keywords:** One Health, CTX-M-producing *E. coli*, antibiotic resistance

## Abstract

CTX-M-producing *Escherichia coli* represents a significant global source of infections, and mitigating its rising prevalence demands a comprehensive understanding of its reservoirs and cross-sectoral distribution. To investigate this, we conducted a One Health surveillance study in Hong Kong (2018–2023), performing a cross-sectoral genomic comparison of CTX-M-producing *E. coli*. A total of 3,213 samples were collected, of which 397 tested positive, including ready-to-eat foods (0.6%, 13/2,119), raw meats (30.0%, 232/774), 217 animal feces (66.4%, 144/217), and food-processing surfaces (7.8%, 8/103). Genomic analysis was performed on all isolated CTX-M-producing *E. coli* strains, which were compared with human clinical isolates. Our results revealed a diverse genomic population of CTX-M-producing *E. coli*, with most isolates clustering in sector-specific groups, suggesting limited cross-sectoral transmission. In contrast, the *bla*_CTX-M-14_ and *bla*_CTX-M-55_ variants were detected across all sectors exhibiting broader dissemination. Dissemination of *bla*_CTX-M_ across these sectors was primarily mediated by transposons (notably Tn*Ecp1.1* and Tn*6339*, driven by IS*Ecp1*), with plasmids playing a secondary role. However, clonal dissemination of *bla*_CTX-M_ between human and non-human sectors was rare. These findings delineate the primary mode of *bla*_CTX-M_ spread and provide a novel assessment to refine antimicrobial resistance surveillance strategies.

**IMPORTANCE:**

The rising prevalence of CTX-M-producing *Escherichia coli* poses a severe global threat to public health by undermining the effectiveness of essential antibiotics. Our comprehensive One Health study provides a critical breakthrough by identifying the primary mechanism behind the spread of the *bla*_CTX-M_ gene. Our research demonstrates that the critical antibiotic resistance gene *bla*_CTX-M_ spreads across human, animal, and food origins not primarily through the transmission of the bacteria themselves, but through mobile genetic elements called transposons. This is a pivotal distinction for public health. It means that surveillance efforts must expand beyond tracking bacterial outbreaks to specifically monitor these mobile gene carriers. By identifying transposons as the main drivers of cross-sectoral spread, our work provides a new and more effective target for strategies aimed at containing the global threat of antimicrobial resistance.

## INTRODUCTION

The prevalence and propagation of antimicrobial resistance (AMR) are serious global public health concerns, with a notable increase in extended-spectrum beta-lactamase (ESBL)-producing *Enterobacterales* in recent years (1, 2). Among these, *Escherichia coli* is the most prevalent pathogen, and CTX-M-type ESBLs have become the predominant beta-lactamases, surpassing TEM and SHV types since the early 21st century (3). Furthermore, driven by mobile genetic elements (MGEs), *bla*_CXT-M_ genes have rapidly disseminated across bacterial species (2), facilitating horizontal gene transfer and amplifying resistance (4). However, the clinical origins and transmission dynamics of these resistant strains remain poorly characterized, hindering effective strategies to mitigate their spread.

The One Health framework, which integrates humans, animal, food, and environmental surveillance, provides a holistic approach to studying AMR (5). This paradigm is particularly relevant for tracking the evolutionary origins and cross-sectoral transmission pathways of clinically relevant AMR. A One Health study was conducted to compare *bla*_CTX-M_ gene transfer between patients, their animals, and related environment (6). Potential sources of human infection or colonization include food-producing animals (7), wastewater (8), and contaminated environments (9). Transmission may occur via the food chain, direct human-animal contact, or exposure to fecal-polluted water and soil, particularly in low- and middle-income countries (10). However, a study from Hanoi, Vietnam, found no evidence of clonal transmission between humans and food animals due to the lack of shared plasmids carrying antibiotic resistance genes, though the IS6-flanked IS*Ecp1-bla*_CTX-M_-orf477/IS*903B* structure was common across habitats (11). These contradictory findings underscore the urgent need for expanded, multi-sectoral surveillance to elucidate *bla*_CTX-M_ gene flow and guide targeted interventions.

Hong Kong’s unique urban ecosystem, which is characterized by exceptional population density and intensive human-animal interactions in wet markets, presents an ideal setting for studying AMR dissemination dynamics (12). The rising incidence of human infections caused by CTX-M-producing *E. coli* in Hong Kong (13, 14) also demands systematic investigation of potential reservoirs. Existing studies implicate multiple potential sources of CTX-M-producing *E. coli*, including retail meat (15–17) and peridomestic rats (18), yet comprehensive studies examining *bla*_CTX-M_ transmission across One Health sectors remain scarce. A deeper understanding of these dynamics would help identify potential sources and transmission routes of CTX-M-producing *E. coli*.

Utilizing a One Health approach, we conducted a cross-sectoral comparison of genetic populations, sharing of *bla*_CTX-M_, and associated MGEs to enhance our understanding of the dissemination model of *bla*_CTX-M_ genes.

## RESULTS

### Prevalence of CTX-M-producing *E. coli*

During September 2018 to August 2023, a total of 445 CTX-M-producing *E. coli* isolates were collected, including 397 from non-human sources (232 raw meat, 13 ready-to-eat [RTE] food, 144 animal fecal samples, and 8 food-processing surface samples) and 48 from human blood samples (Table S1).

Among raw meat samples, 232 CTX-M-producing *E. coli* were recovered, with fresh meat showing significantly higher contamination rates than chilled meat (*P* < 0.00001, chi-square test; Table S2). Animal fecal samples exhibited striking differences in prevalence, with chicken feces showing the highest rate (86.0%, 86/100), followed by pigs (59.7%, 40/67) and cattle (36.0%, 18/50). In contrast, CTX-M-producing *E. coli* was uncommon on food-processing surfaces (7.8%, 8/103) and in RTE foods (0.6%, 13/2,119).

### Cross-sectoral population structure and *bla*_CTX-M_ diversity

**Fig 1:**
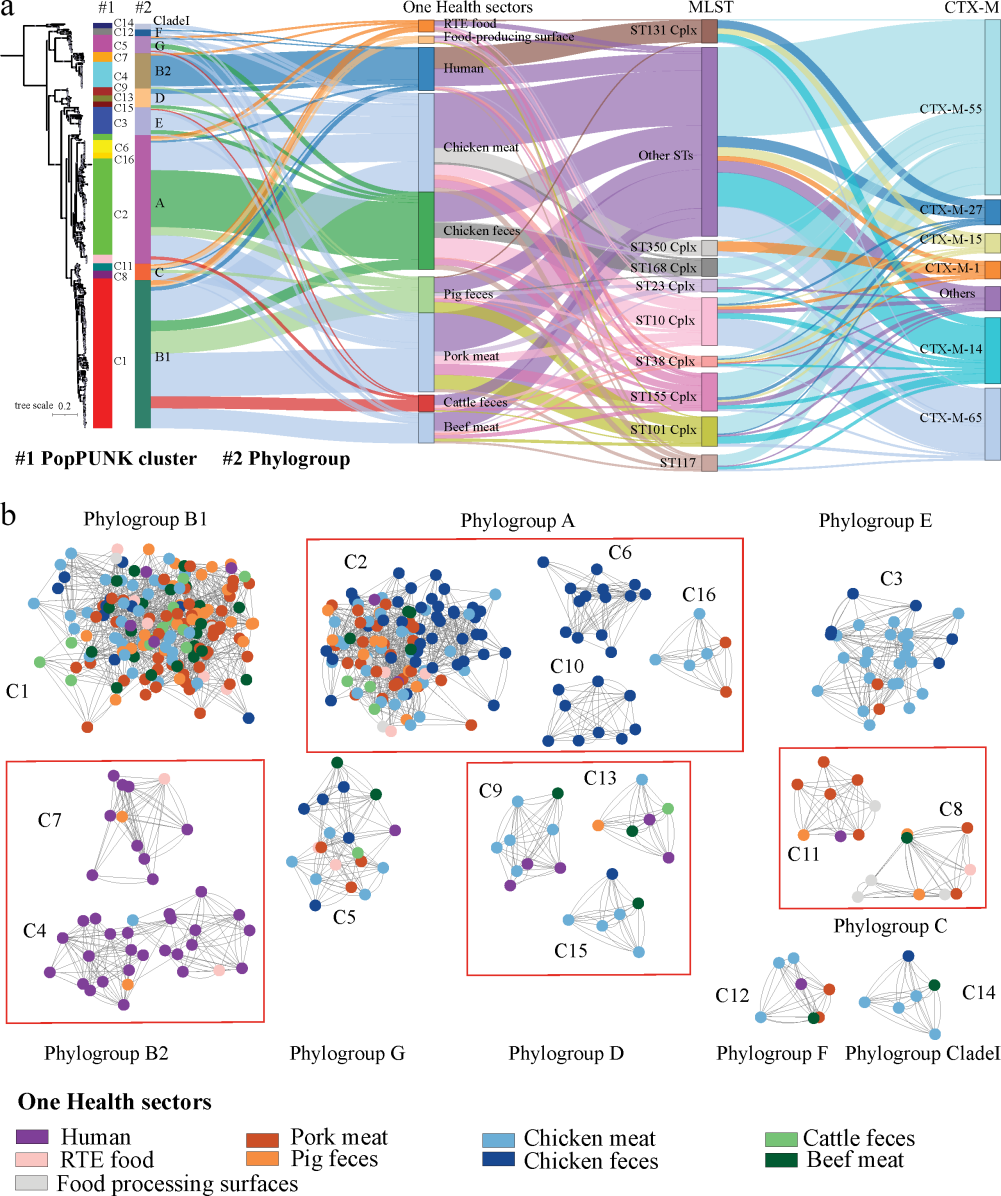
Population structure of our CTX-M-producing *E. coli*. (**a**) A mid-rooted, maximum-likelihood phylogenetic tree of 445 CTX-M-producing *E. coli* isolates was aligned with metadata including PopPUNK cluster, phylogroup, One Health sectors, MLST, and CTX-M alleles. The circle size on each branch corresponds to bootstrap values, ranging from 90 to 100. Only MLST and CTX-M alleles representing at least 10 genomes within the data set are displayed individually. The remaining genomes are grouped as others. (**b**) The PopPUNK network analysis for 445 CTX-M-producing *E. coli* genomes labeled by their respective cluster. The nodes were colored by One Health sectors. Clusters that belong to the same phylogroup are indicated by a red rectangle.

We established a core genome phylogenetic tree of the 445 CTX-M-producing *E. coli* isolates using 288,016 single-nucleotide polymorphisms (SNPs) to investigate genetic relationships (Fig. 1a). The population structure was highly diverse, comprising 137 STs grouped into 26 clonal complexes (CCs) (Fig. 1a; Table S3). A substantial proportion of isolates (39.3%) were singleton, predominantly from non-human sectors. The most prevalent CCs were sequence type 10 (ST10) complex (Cplx, *n* = 53), ST155 Cplx (*n* = 42), and ST101 Cplx (*n* = 33), primarily from animal and raw meat samples, with ST10 Cplx notably absent in human blood isolates. Conversely, ST131 Cplx dominated human bloodstream isolates (50%, 24/48). PopPUNK analysis identified 16 distinct clusters, with median SNP distances of 37,190 between clusters and 11,852 within clusters (Fig. S1). The largest cluster, C1 (*n* = 164), belonging to phylogroup B1, consisted mainly of non-human isolates with the exception of four bloodstream isolates (Fig. 1b). Chicken fecal isolates exclusively formed clusters C6 and C10, while human blood isolates primarily clustered in C4 and C7, all of which belonged to phylogroup B2.

A total of 130 ARGs were identified across all collected genomes, with 37 of these genes being widely distributed, appearing in over 10% of the total samples. There was a notable co-occurrence of *bla*_TEM_ genes alongside *bla*_CTX-M_. In addition to beta-lactam resistance, other ARGs with high prevalence (detected in over 50% of all samples) included *aph* genes conferring resistance to aminoglycosides, *floR* for phenicol resistance, *gyr* mutations for fluoroquinolone resistance, *sul* for sulfonamide resistance, and *tet(A)* for tetracycline resistance (Fig. S2). The distribution of *bla*_CTX-M_ genes varied significantly across One Health sectors (Fig. S3). Human bloodstream isolates primarily carried *bla*_CTX-M-14_ and *bla*_CTX-M-27_, while non-human isolates predominantly harbored *bla*_CTX-M-55_ and *bla*_CTX-M-65_. Notably, except for a single *bla*_CTX-M-1_-positive isolate from pork meat, all *bla*_CTX-M-1_ genes were detected in imported chilled chicken meat (Table S3). Hybrid *bla*_CTX-M_ genes were exclusively detected in food samples, including beef (*n* = 1), chicken (*n* = 5), pork (*n* = 1), and RTE food (*n* = 2) (Fig. S3). Among these, *bla*_CTX-M-123_ was the most common hybrid variant, detected in four chicken isolates and one pork isolate. *bla*_CTX-M-199_ and *bla*_CTX-M-64_ were carried by two isolates from sushi and Lo Mei, respectively.

### Source prediction and sharing of CTX-M-producing *E. coli*

**Fig 2:**
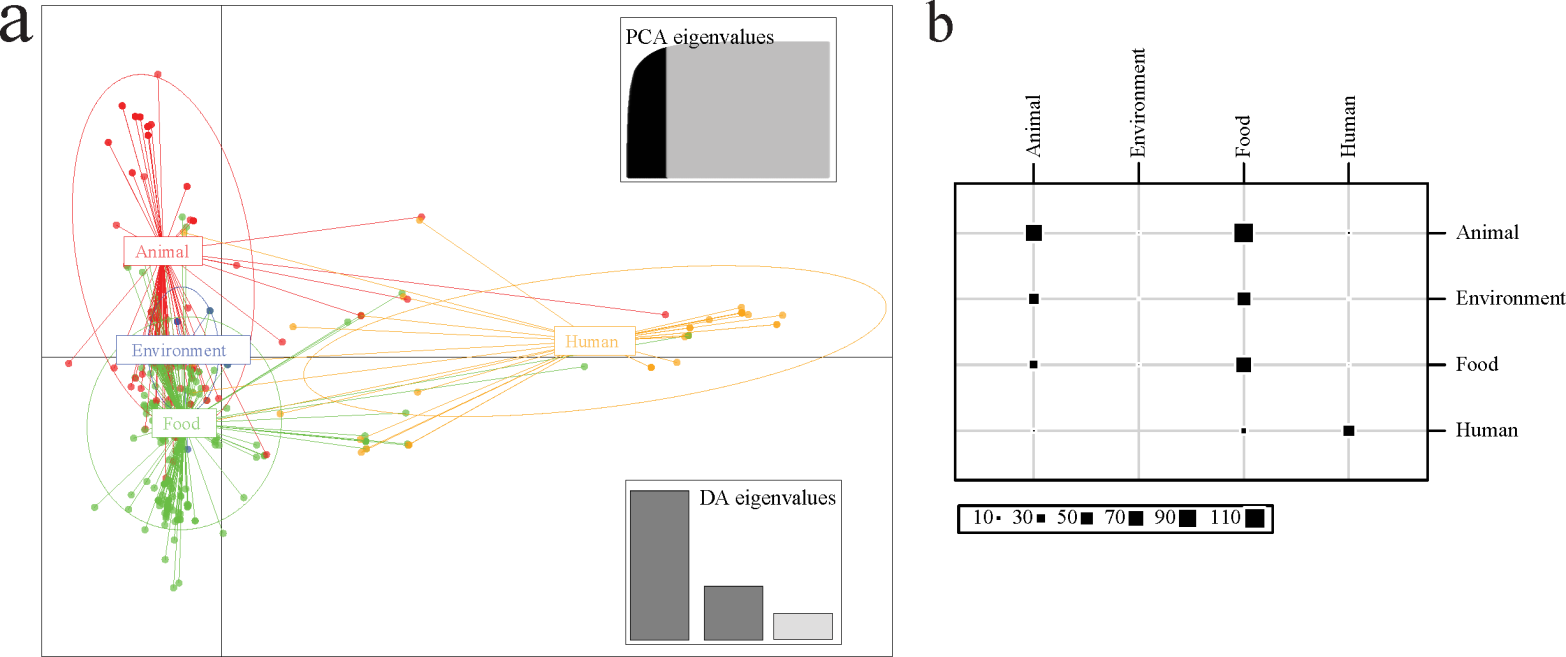
Source trace of CTX-M-producing *E. coli* isolates generated using a Discriminant Analysis of Principal Component (DAPC) detectable model. (**a**) Scatterplot of our CTX-M-producing *E. coli*. Each dot represents one individual isolate, with different origins indicated by different colors. The ellipse indicates the 95% CI for each origin. (**b**) Table plot of the prediction set. Rows and columns represent reported and predicted origins, respectively, while the size of each square indicates the number of corresponding isolates.

Discriminant analysis of principal components (DAPC) was performed to explore relationships of our CTX-M-producing *E. coli* isolates. The reference model, deduced from 1,218 publicly available genomes spanning animal (*n* = 392), environmental (*n* = 334), food (*n* = 165), and human (*n* = 327), indicated close genetic relationships among isolates from animal, environment, and food origins, while human isolates form a separate cluster (Fig. 2a). Source attribution modeling predicted that 42.4% (104/245), 23.7% (58/245), and 5.3% (13/245) of food isolates showed the highest genetic affinity to animal, environment, and human sources, respectively. In contrast, only 2.7% (4/144) of animal isolates were most closely assigned to human-associated genomes (Table S4; Fig. 2b). Surface environment-derived isolates predominantly clustered with animal and food-associated genomes, with none showing the highest affinity to human-associated genomes. Additionally, 83.3% (40/48) of human-derived isolates were assigned to human-associated reference clusters, indicating consistent host-associated genomic structuring within the model.

**Fig 3:**
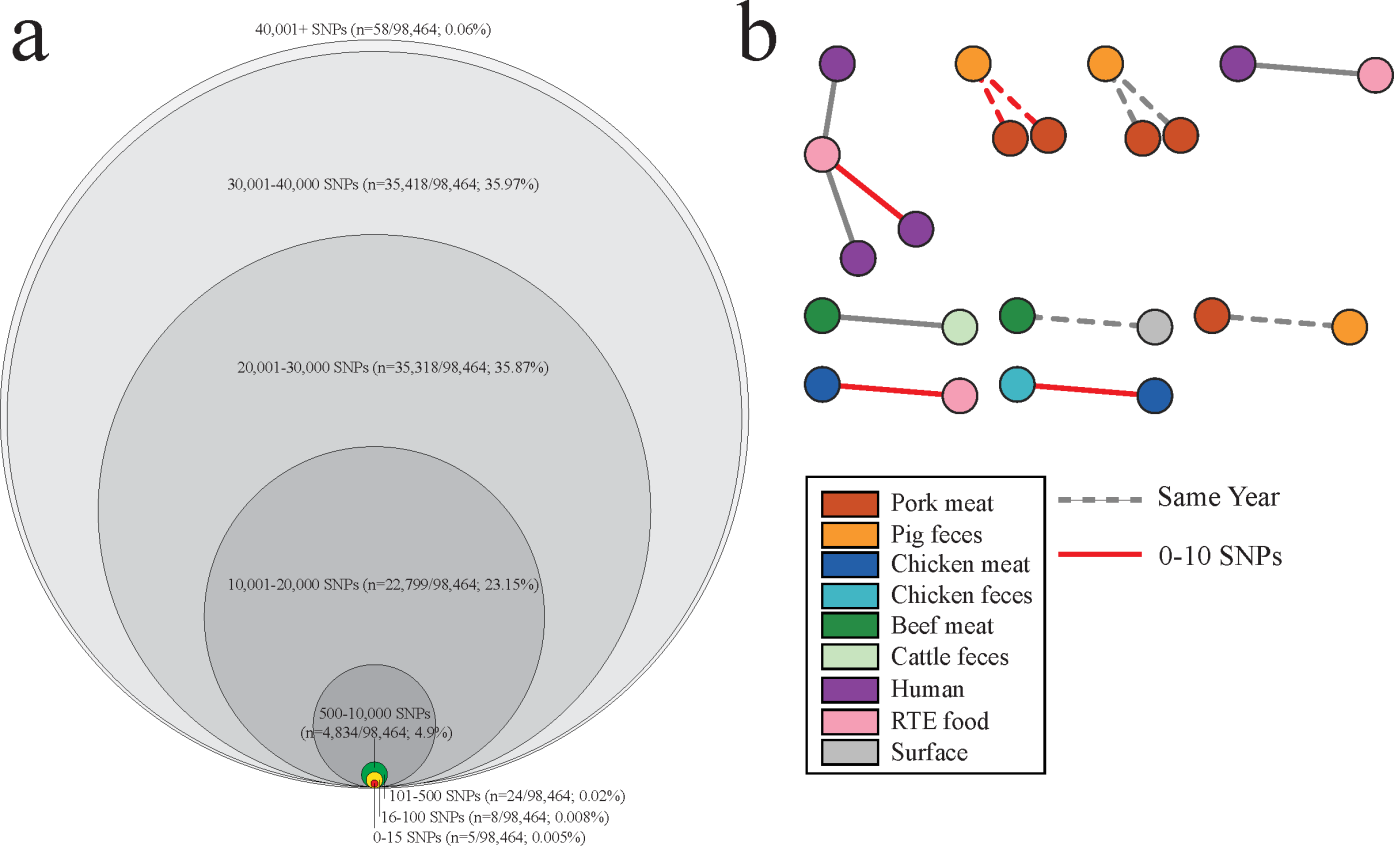
An SNP-based analysis of the clonal dissemination of CTX-M-producing *E. coli* across One Health sectors. (**a**) The Euler diagram shows the frequency of SNP distance among cross-sectoral genome pairs. (**b**) The network shows the cross-sectoral linkage with SNP distance less than 100. The color of the node indicates the One Health sector. Nodes shared less than 10 SNPs are indicated with a red line. Dashed lines indicate samples collected within the same year, whereas solid lines indicate samples collected in different years.

Pairwise SNP distance analysis among cross-sectoral isolates revealed that the majority of pairs exhibited over 20,000 SNPs (Fig. 3a), with a median SNP distance of 18,296 in food-processing surface isolates, whereas other sources ranged from 21,277 to 34,740 (Fig. S4). Among all pairs, only 37 pairs (<0.1%) displayed fewer than 500 SNPs. Pairs (<500 SNPs) between human and non-human genomes predominantly originated from RTE food and chicken meat samples, with *bla*_CTX-M-55_, *bla*_CTX-M-27_, and *bla*_CTX-M-65_ being frequently shared (Table S5). For a quantitative assessment of cross-sectoral sharing, we defined cross-sectoral linkage as those with ≤100 SNP differences, identifying only 13 such pairs comprising a total of 22 isolates (Fig. 3b). Only two RTE food items (RTE vegetables and sushi) were predicted to have cross-sectoral linkages with human-associated genomes, with one pair exhibiting fewer than 10 SNPs. Additionally, two genomes from chicken meat displayed fewer than 10 SNPs when compared with isolates from chicken feces and RTE food. Notably, all cross-sectoral linkages observed in pig samples were from the same year.

### Sharing of *bla*_CTX-M_-associated plasmids

Our analysis of 493 *bla*_CTX-M_ genes from the 485 genomes revealed 19 distinct *bla*_CTX-M_ alleles (Fig. S5; Table S3), with *bla*_CTX-M-14_ and *bla*_CTX-M-55_ appearing across all One Health sectors (Fig. S6). Pig-associated samples carried five of seven *bla*_CTX-M_ alleles shared between human and non-human sources. Geographically, most *bla*_CTX-M_ sharing was observed between Mainland China and Hong Kong, except for *bla*_CTX-M-1_, which was exclusively detected in regions outside China, predominantly in Europe.

**Fig 4:**
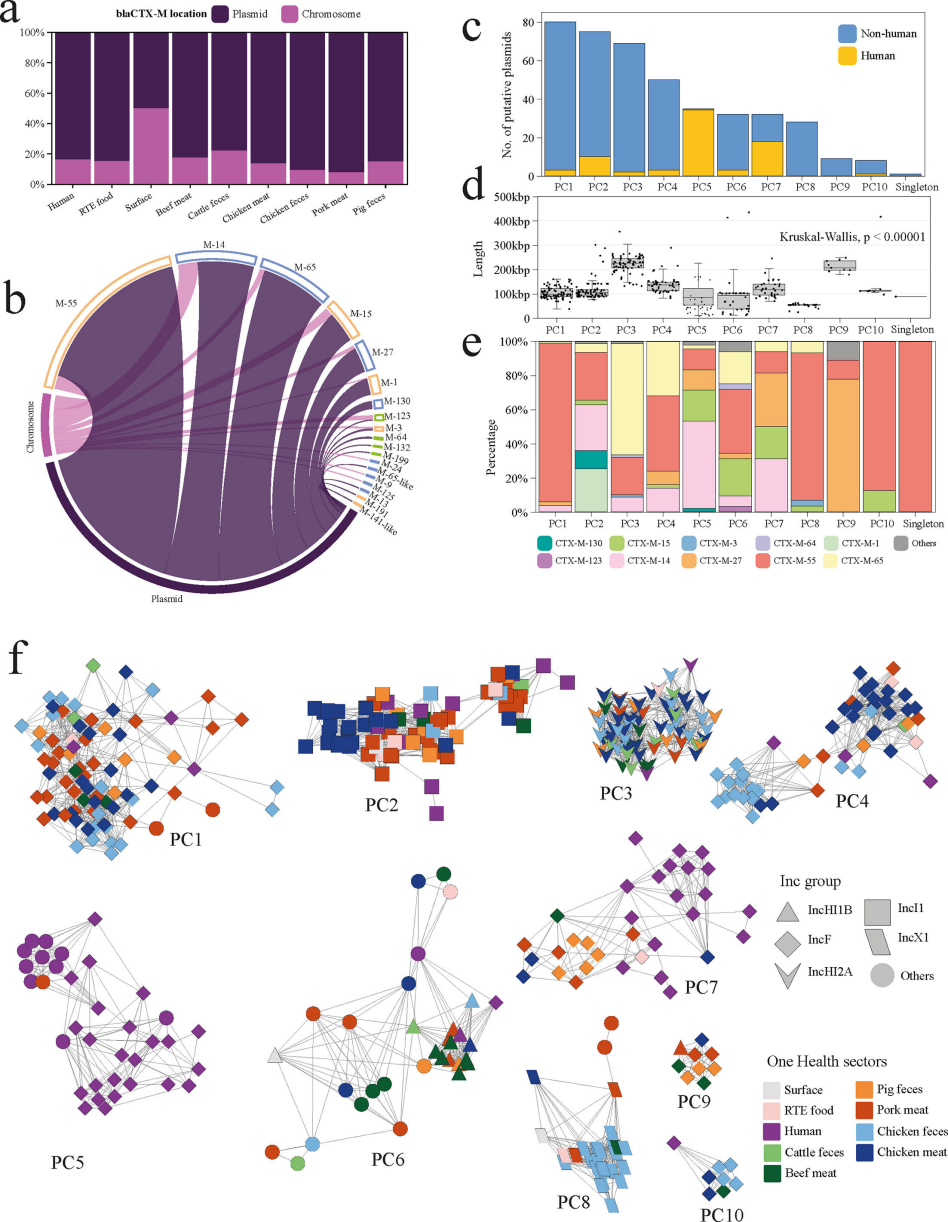
Putative *bla*_CTX-M_-carrying plasmids across One Health sectors. (**a**) The stacked bar plot shows the location of *bla*_CTX-M_ across One Health sectors. (**b**) Circus diagram shows the distribution of *bla*_CTX-M_ genes in the plasmid and chromosome. (**c**) The bar plot illustrates the number of putative plasmids within each cluster. (**d**) The box plot presents a comparison of the size of plasmids in each cluster. Boxes represent 50% of the central data, in between the lower and upper quartiles, with a central line representing the median. Statistical analysis was performed using the Kruskal-Wallis test. (**e**) Distribution of *bla*_CTX-M_ across plasmid clusters. (**f**) Network visualization of *bla*_CTX-M_-carrying plasmid clusters (PCs). Nodes are color-coded according to One Health sectors, and the shape of nodes indicates the Inc type of plasmids.

To assess the potential transferability of *bla*_CTX-M_ across One Health sectors, we characterized their associated putative plasmids. Chromosomal localization was uncommon (<20% of isolates in each sector) except in food-processing surface samples (~50%) (Fig. 4a). All *bla*_CTX-M_ genes were plasmid borne, with the exception of *bla*_CTX-M-123_, which was primarily chromosomal borne (Fig. 4b). The 419 putative plasmids were classified into 10 plasmid clusters (PCs) based on pangenome similarity, including one singleton (Fig. 4c). Human-derived plasmids predominated in PC5 and PC7, whereas clusters PC8 and PC9 consisted exclusively of non-human plasmids. Plasmid size varied significantly across clusters (*P* < 1e-5, Kruskal-Wallis test), averaging 152,500 ± 98,014 bp (Fig. 4d), with the largest average size at 316,939 ± 147,347 bp observed in PC5 (primarily human derived). Most clusters (PC1, PC8, and PC10) from non-human origins carried mainly *bla*_CTX-M-55_, whereas *bla*_CTX-M-65_ dominated in PC3. Human and non-human sharing clusters (PC2 and PC7) showed high allele diversity, with frequent *bla*_CTX-M-14_ and *bla*_CTX-M-55_ identified (Fig. 4e).

Replicon typing revealed IncF (49.8%, 215/432), IncI1, IncHI2A, IncX1, and IncHIB as predominant types (Table S3). Inc types represented by fewer than 10 plasmids were categorized as “others.” IncF plasmids were widely distributed across clusters PC1, PC4, PC5, and PC7 (Fig. 4f), whereas other Inc types showed cluster specificity, such as IncI1 in PC2, IncHI2A in PC3, and IncX1 in PC8. Non-human genomes dominated IncF-rich clusters PC1 and PC4, and IncHI2A-rich PC3. Notably, among clusters PC2 and PC7 shared between human and non-human sources, PC2 was exclusively composed of IncI1 plasmids, with those carrying *bla*_CTX-M-14_ and *bla*_CTX-M-55_ being notably shared between human and pig hosts (Table S3).

### Sharing of *bla*_CTX-M_-associated mobile genetic islands

**Fig 5:**
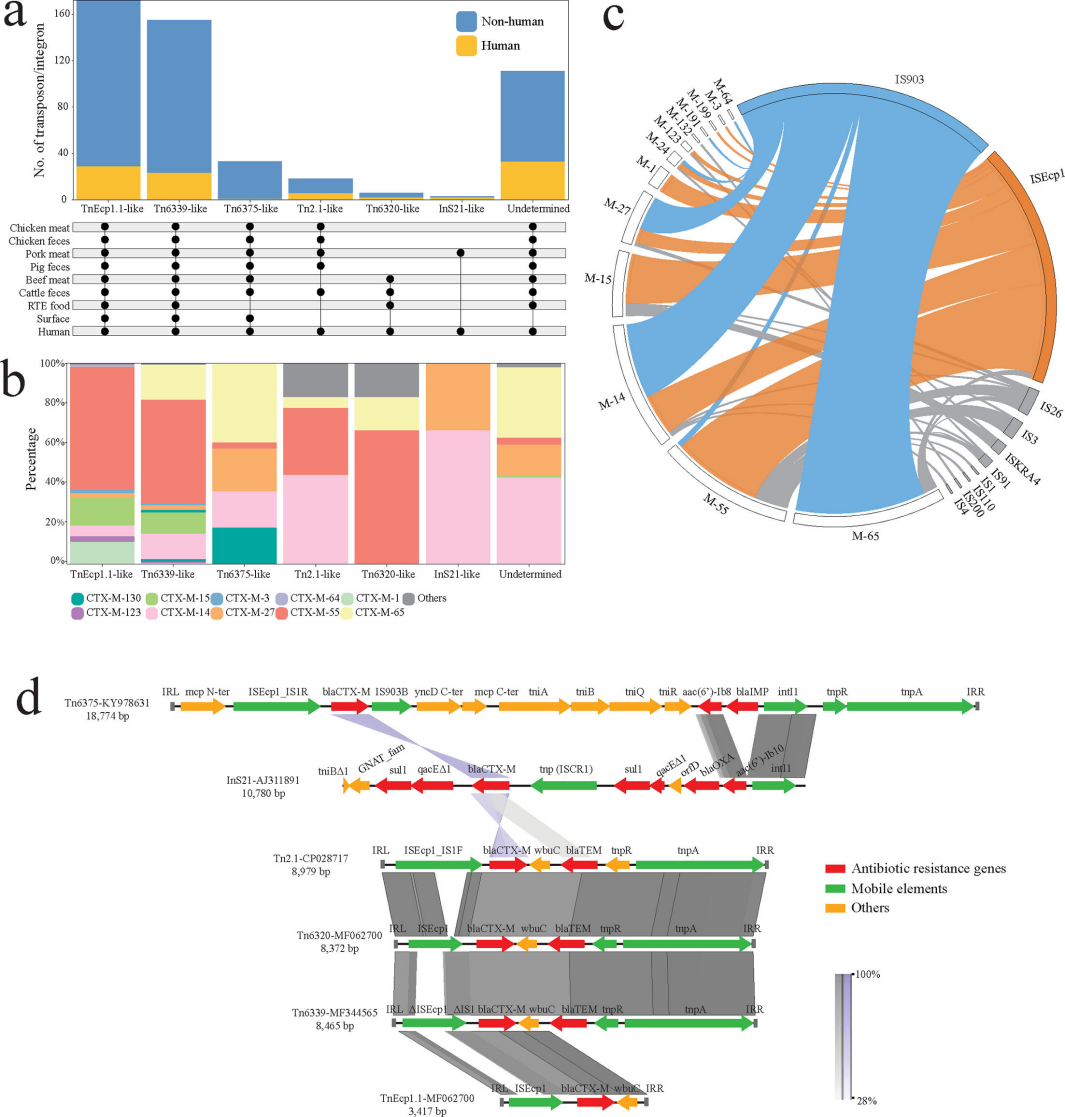
Cross-sectoral *bla*_CTX-M_-associated mobile genetic elements. (**a**) The stacked bar chart illustrates the number of identified transposon/integron (Tn/In) elements. The lower upset plot indicates the sharing of Tn/In across each One Health sector, as demonstrated by the presence of nodes. (**b**) The stacked bar chart displays the distribution of *bla*_CTX-M_ alleles across different Tn/In elements. (**c**) The circus diagram depicts the distribution of *bla*_CTX-M_ genes in relation to adjacent IS elements. (**d**) The genetic alignment of six identified Tn/In elements is shown, with arrows colored to represent different functional categories.

To further characterize the mechanisms facilitating cross-sectoral dissemination of *bla*_CTX-M_ genes, we analyzed their surrounding genetic architectures, including associated insertion sequences (ISs), transposons (Tn), and integrons (In). A total of six Tn/In elements were identified for *bla*_CTX-M_ dissemination, with the unidentified elements primarily flanked by IS*903*, followed by IS*Ecp1* (Fig. S7). Among all *bla*_CTX-M_-associated genetic architectures, Tn*Ecp1.1*- and Tn*6339*-like elements were detected across all One Health sectors. Although Tn*6375*-like elements also exhibited extensive cross-sectoral dissemination, most were derived from non-human sources, with only a single occurrence in a human isolate genome (Fig. 5a; Table S6). Regarding *bla*_CTX-M_ allele distribution, Tn*Ecp1.1*- and Tn*6339*-like elements were prevalently associated with *bla*_CTX-M-55_, followed by *bla*_CTX-M-15_. Infrequent elements, Tn*6375*-, Tn*2.1*-, Tn*6320*-, and In*S21*-like, were associated with *bla*_CTX-M-55_ and *bla*_CTX-M-14_. *bla*_CTX-M-1_ was detected exclusively in Tn*Ecp1.1* and *bla*_CTX-M-65_ in Tn*6339* (Fig. 5b). For these transposons, 11 *bla*_CTX-M_-associated ISs were identified, with IS6 family IS*903* and IS*Ecp1* as the most prevalent (Fig. 5c; Table S7), which were commonly observed in IS*Ecp1-bla*_CTX-M_-IS*903*/*wbuC* mobile genetic islands within identified transposons, and also for those undetermined genetic structures (Fig. 5d). These mobile genetic islands were prevalently associated with *bla*_CTX-M-55_ and *bla*_CTX-M-14_ and shared cross sources, between human and non-human origins.

### Reconstructing the mode of cross-sectoral *bla*_CTX-M_ dissemination

**Fig 6:**
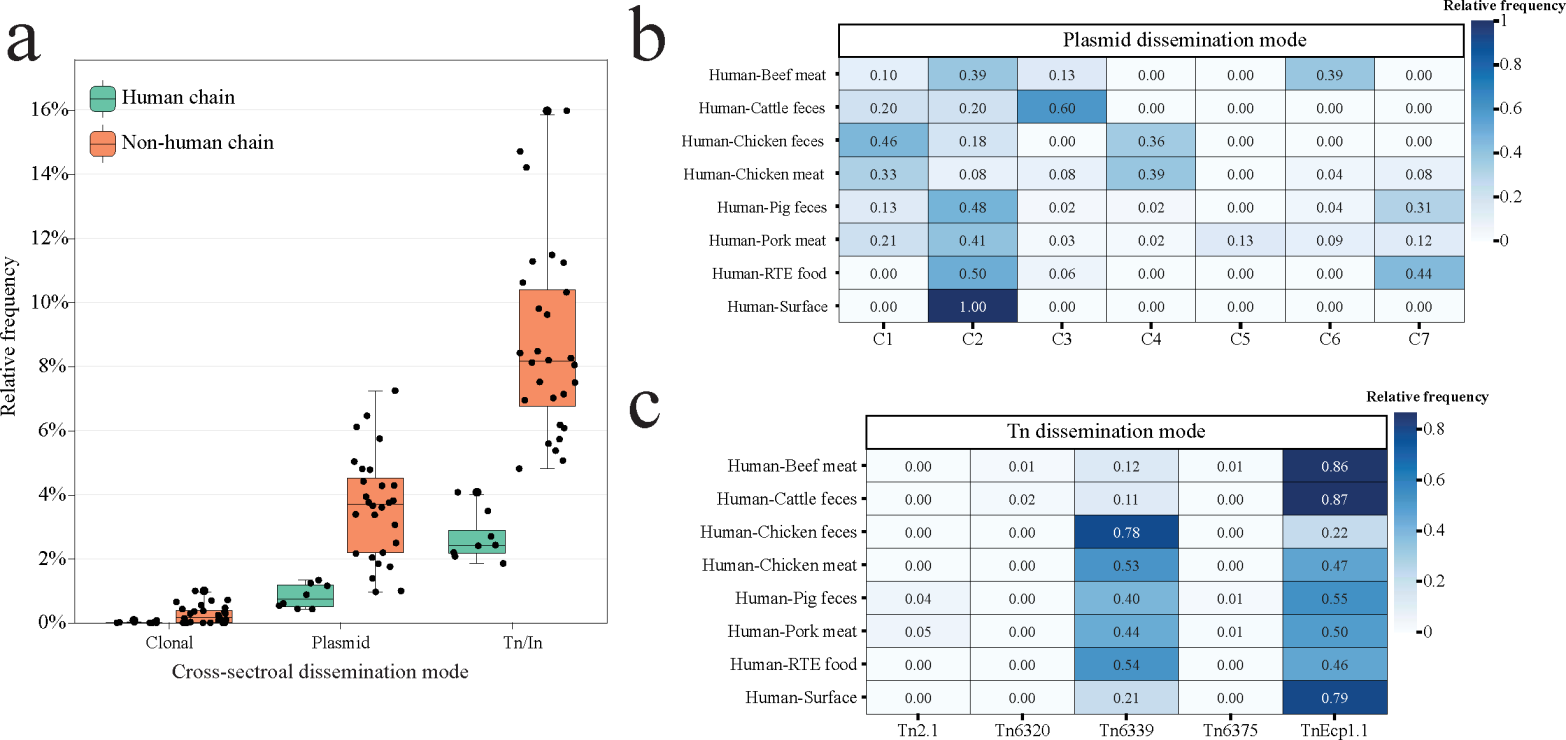
Contribution of dissemination modes in the cross-sectoral spread of *bla*_CTX-M_ gene. (**a**) The relative frequency of dissemination modes for each pairwise cross-sector comparison is shown. Cross-sectors are categorized into human and non-human chains based on the origins of the isolates. In the box plots, the boxes represent the interquartile range, covering the central 50% of the data, with the central line indicating the median. (**b**) The heatmap displays the frequency of plasmid dissemination modes across each pairwise sector within the human chain. (**c**) The heatmap illustrates the frequency of transposon/integron (Tn/In) dissemination modes across each pairwise sector within the human chain.

From a One Health perspective, our findings suggest that clonal expansion played a minor role in *bla*_CTX-M_ dissemination, with an average frequency of less than 2% (Fig. 6a; Table S8). Instead, horizontal gene transfer via MGEs was the dominant mechanism. In the non-human chain, *bla*_CTX-M_ cross-sectoral dissemination occurred more frequently, primarily mediated by Tn transfer. Plasmid-mediated dissemination was accentuated in PC2 detected in all human chains (Fig. 6b). For plasmids in PC1 and PC4, we observed a high frequency of plasmid spread between human isolates and chicken isolates. Besides, PC3 plays a key role in *bla*_CTX-M_ dissemination between human and cattle feces with a frequency of 0.6. Regarding mobile genetic islands, we observed no sharing of the same In*S21*-like element between human and non-human origins. The dissemination mode was primarily associated with Tn*Ecp1.1* and Tn*6339*; specifically, Tn*Ecp1.1* was mainly linked between humans and cattle, whereas Tn*6339* was predominantly associated with chickens, particularly in chicken feces (Fig. 6c).

## DISCUSSION

The persistent emergence of CTX-M-producing *E. coli* poses significant treatment challenges in both human and veterinary medicine because of their widespread dissemination across One Health sectors. This proliferation is particularly evident in animal populations, where intensive antibiotic use in livestock production has been identified as a major contributing factor (19). In chicken feces, *E. coli* isolates exhibit low genetic diversity, with a high level of within-sector isolate sharing observed in Hong Kong. The primary explanation for this observation is that all chicken fecal samples were collected in Hong Kong, while the cattle and pig fecal samples came mainly from animals imported from mainland China. This regional restriction is a significant limitation for cross-sectoral clonal expansion, corresponding with previous observations that the diversity of ARGs from different regions (20). Several studies have reported the hybrid CTX-M genes were detected from humans (21), wastewater (22), and animals (23). Notably, this study is the first to report the presence of hybrid *bla*_CTX-M_ genes in food samples, specifically *bla*_CTX-M-123_, *bla*_CTX-M-199_, and *bla*_CTX-M-64_. It is hypothesized that these hybrid *bla*_CTX-M_ genes are disseminated across hosts via IS*Ecp1*-mediated transposition. Although our analysis could not elucidate the evolutionary pathways of *bla*_CTX-M_ hybrid genes, previous studies (24, 25) suggest that frequent transmission mediated by IS*Ecp1* facilitates the formation of these hybrid genes. Moreover, the detection of CTX-M-producing *E. coli* on food-processing surfaces suggests that transmission through the food chain is possible. However, it is important to note that such clonal dissemination remains infrequent. These findings emphasize the vital importance of maintaining strict food hygiene practices to prevent contamination and protect public health (26, 27).

The widespread prevalence of CTX-M-producing *E. coli* in the food chain has led to ongoing debate regarding its transmission pathways to humans, but clarification of this is critical to develop mitigation strategies. Numerous studies have employed an SNP distance threshold of <100 to detect the cross-sectoral linkage and standardize the parameters for One Health genomic surveillance (28–30). However, a limited connection between human infection isolates and those from livestock was observed in several regional studies (10, 31). Similar to previous analyses (10, 11, 31, 32), the genomic population of CTX-M-producing *E. coli* demonstrates that isolates from animal feces and raw meat were not closely related isolates causing human bloodstream infection. CTX-M-producing *E. coli* from non-human origins exhibited a diverse array of clusters, and the majority of *E. coli* sharing occurs within sectors with regional specificity, whereas *E. coli* phylogroup B2 has been frequently implicated in human infections, as previously reported (10, 31). Several instances of cross-sectoral isolate sharing were observed with ST117 *E. coli*, which showed an SNP distance of zero and was present in humans, RTE food, and animals. Previous research has documented the zoonotic potential of ST117 *E. coli*, noting its co-occurrence in humans, animals, the environment, and food (33). Consistent with earlier findings, the observed sharing of ST117 *E. coli* isolates in Hong Kong indicates the potential for cross-sectoral transmission.

The *bla*_CTX-M_ distribution varied across different One Health sectors. Notably, *bla*_CTX-M-1_/IncI1-positive *E. coli* was primarily isolated from chilled chicken meat imported from the UK, with no detection from local isolates in Hong Kong. *E. coli* producing the CTX-M-1 group has been found to be widely and dominantly disseminated in the UK, with *bla*_CTX-M-15_ being particularly common (34). Additionally, the *bla*_CTX-M-1_ gene is frequently reported in other European regions, such as Germany (35) and Norway (36), yet there are limited reports of its presence in Asia (2). Consequently, the identification of *bla*_CTX-M-1_ underscores the need for enhanced prevention strategies and further screening efforts to strengthen potential *bla*_CTX-M-1_-positive *E. coli* transmission routes. Consistent with previous analyses of CTX-M-producing *E. coli* from humans and animals (10, 11), *bla*_CTX-M-14_ and *bla*_CTX-M-15_ were the main alleles identified in humans, whereas *bla*_CTX-M-55_ and *bla*_CTX-M-65_ were dominant in non-human *E. coli* genomes. Despite the dominance of different *bla*_CTX-M_ alleles in human and non-human isolates, *bla*_CTX-M-14_ and *bla*_CTX-M-55_ were detected across all One Health sectors, indicating a significant potential for cross-sectoral horizontal gene transfer. CTX-M-producing *E. coli* from pig hosts displayed a complete overlap with all human *bla*_CTX-M_ genes. A strong intersection was also observed in a prior study, which reported a closer genetic distance of ARGs from human hosts in Hong Kong and swine hosts in China (20). Furthermore, our study is the first to report a *bla*_CTX-M_ hybrid gene in a food item. These findings underscore the potential risk of *bla*_CTX-M_ sharing between animals, food, and humans, highlighting the need to inform strategies aimed at controlling the cross-sectoral dissemination of ARGs.

To further explore cross-sectoral sharing, we propose that frequent shifts in the *bla*_CTX-M_ gene may occur through identical or closely related MGEs. Previous studies have shown that *bla*_CTX-M-14_ can be located on various plasmid types, including IncF, IncI1, IncA/C, IncN, and IncK2 (37, 38), predominantly in isolates from animal-related sources (2). The high variability of *bla*_CTX-M-14_-carrying plasmids may be a key factor facilitating the gene shift from non-human to human isolates. Similarly, *bla*_CTX-M-55_ is widely detected across different hosts, primarily associated with IncI1 and IncI2 plasmids (2). Our study extended these observations by identifying several instances of *bla*_CTX-M_-associated MGE sharing in C2/IncI1 plasmids and C7/IncF plasmids. Furthermore, we identified frequent sharing of Tn*3* family transposons facilitating *bla*_CTX-M_ dissemination. The key transposons, Tn*6339* and Tn*Ecp1.1*, play a dominant role in *bla*_CTX-M_ spread between human and non-human origins. This finding bridges the gap in previous research, which observed distinct plasmids but identical *bla*_CTX-M_ between humans and livestock (11, 31). Notably, the transmission of *bla*_CTX-M_-associated Tn appears to be primarily mediated by IS*Ecp1* and IS*903*, which are located upstream and downstream of the gene. To elucidate the mode of *bla*_CTX-M_ spread, we constructed a dissemination scenario based on previous studies tracking vancomycin resistance in the Netherlands (39). The analysis of dissemination modes was initially developed to track the dynamics of ARG transmission across different time frames within hospitals. To apply this method within a One Health context, we employed the same strategy used to define dissemination modes in human and non-human origins. These analyses validate and generalize the findings that clonal dissemination is infrequently detected between human and non-human origins; however, highly similar plasmids and transposons can be transferred between different clonal complexes. These findings highlight the interconnected nature of CTX-M-producing *E. coli* concerning *bla*_CTX-M_-associated MGEs, offering valuable insights into strategies for controlling and mitigating the spread of *bla*_CTX-M_ genes across One Health sectors.

Our study has several limitations. First, the DAPC analysis was based on public genomes derived from heterogeneous sources. As DAPC is sensitive to sampling bias, this heterogeneity may lead to overestimation or misrepresentation of genomic affinity across One Health sectors. Due to the disruption of MGEs by short-read sequencing and the prohibitive cost of long-read sequencing for all samples, *bla*_CTX-M_-associated MGEs were identified and confirmed using alignment-based methods. However, this approach may overlook novel MGEs. Besides, expanding this collection to include human isolates from urine, sputum, and stool samples would provide a more comprehensive understanding of CTX-M-producing *E. coli* cross-sectoral diversity. In conclusion, this study demonstrates the presence of genetic characteristics of CTX-M-producing *E. coli* across raw meat, RTE food, food-producing surfaces, animal feces, and humans in Hong Kong during 2018–2023. We have not generated strong evidence to suggest that the dominant *E. coli* strains responsible for human infections originate from animal and food origins. However, we observed significant sharing of MGEs between human and non-human *E. coli* genomes. The constructed dissemination mode indicates that transposons, in conjunction with plasmids, are key factors facilitating the cross-sectoral spread of *bla*_CTX-M_.

## MATERIALS AND METHODS

### Sampling

A cross-sectoral survey was performed between September 2018 and August 2023 to isolate CTX-M-producing *E. coli* from food items including 774 raw meat and 2,119 RTE food across all districts in Hong Kong except the Island district. The details of food samples contain 288 beef meat (252 chilled meat and 36 fresh meat), 324 chicken meat (288 chilled meat and 36 fresh meat), 162 pork meat (110 chilled meat and 52 fresh meat), 287 sashimi, 228 sushi, 86 smoked salmon, 269 RTE cut fruit, 227 RTE vegetable salads, 212 Siu Mei, 111 Lo Mei, 359 RTE sandwiches, and 340 RTE oysters. Specifically, only raw fresh meat was purchased from local wet markets in Hong Kong, while other food items were obtained from supermarkets.

During December 2018–March 2021, 217 food-producing animals were sampled at a slaughterhouse, including rectal samples from pigs (*n* = 67), cattle (*n* = 50), and chicken (*n* = 100). All rectal swabs were placed in sterile tubes and then transported to the laboratory weekly for isolation of CTX-M-producing *E. coli*.

Between September 2020 and November 2020, a total of 103 sponge swab samples were taken from surfaces, including cutting boards and weighing scales. All surface swab samples were collected from households and wet market stalls. For samples from households (*n* = 95), the target for sampling was cutting boards, with criteria including that the board had been used for handling raw meat within the past week and had been in use for a minimum of 3 months. For samples from wet market stalls (*n* = 8), samples were taken from cutting boards and weighing scales, with the sole inclusion of item used for processing raw meat.

Furthermore, a total of 88 CTX-M-producing *E. coli* samples from human sources were collected, comprising 48 isolates from bloodstream infections and 40 publicly available *E. coli* genomes isolated from humans in Hong Kong. The public human isolates were collected by searching the NCBI Pathogen Detection database (https://www.ncbi.nlm.nih.gov/pathogens/) using query “geo_loc_name: Hong Kong AND taxgroup_name:" E.coli and Shigella " AND AMR_genotypes:blaCTX-M*.” Based on our sampling strategies, we defined a total of nine One Health sectors for further analysis, including human, RTE food, food processing surfaces, pork meat, pig feces, chicken meat, chicken feces, cattle feces, and beef meat.

### Bacterial culture and microbiological tests

For food samples, each 25 g food sample was placed into a Stomacher 400 Circulator Standard bag (Seward, West Sussex, USA) containing 225 mL of tryptic soy broth (Becton Dickinson, Washington, USA) supplemented with 10 µg/mL vancomycin, 5 µg/mL fluconazole, and 2 µg/mL cefotaxime. These bags were thoroughly mixed using a BagMixer 400 P Stomacher (Interscience, Saint-Nom-la-Bretèche, France) for 3 min and then incubated at 37°C for 16–20 h. Subsequently, 10 μL of this mixture was inoculated onto CHROMID ESBL agar (bioMérieux, Marcy l’Etoile, Lyon, France) using a disposable inoculation loop and incubated at 37°C for 16–20 h. In the case of animal and food-processing swabs, each swab was directly streaked onto CHROMID ESBL agar (bioMérieux) and incubated under the same conditions. Colonies displaying pink to burgundy coloration were transferred to Columbia blood agar plates supplemented with 5% horse blood (Thermo Fisher Scientific, Waltham, MA, USA) and incubated at 37°C for 16–20 h. Subsequently, a pure colony from the blood agar was selected for species verification using the matrix-assisted laser desorption/ionization-time-of-flight mass spectrometry technique (Bruker, Billerica, MA, USA).

The phenotypic detection of ESBL-producing *E. coli* strains was determined by a double-disk synergy test. Antimicrobial susceptibility testing for all presumptive ESBL-producing *E. coli* isolates was conducted using the disk diffusion method in accordance with the Clinical and Laboratory Standards Institute M100-34 guidelines (40). Quality control was assured by including the *E. coli* strain ATCC 25922 and *Pseudomonas aeruginosa* strain ATCC 27853 in each batch of antimicrobial tests. The CTX-M-producing *E. coli* was confirmed by PCR with specific primers (Table S9) for detection of CTX-M-1, CTX-M-2, CTX-M-9, CTX-M-8, CTX-M-25, and hybrid groups.

### DNA sequencing

Genomic DNA extraction was performed on all CTX-M-producing *E. coli* isolates using the DNeasy Blood & Tissue Kit (QIAGEN, Venlo, Netherlands). Library preparation was conducted according to the Illumina protocol using the NEBNext Ultra DNA Library Prep Kit (New England Biolabs, Ipswich, MA, USA). Then, all library mixtures were subjected to short-read sequencing using a NovaSeq 6000 system with a 2 × 150 bp paired-end protocol.

### Genome assembly and annotation

All raw reads were trimmed to eliminate adapters using Trimmomatic v.0.39 (41). Paired-end reads were then assembled using SPAdes v.3.15.5 (42) with --careful flag for precision. Assembly statistics were calculated using QUAST v.5.2.0 (43). Taxonomic identification on the strain level was performed using GTDB-Tk v.2.3.2 with the r214 database (44), and CheckM v.1.2.1 (45) was performed to assess genomic quality. All genomes were annotated with Prokka v.1.14.6 (46) with default parameters. For annotating ARGs, NCBI Antimicrobial Resistance Gene Finder (AMRFinderPlus) (47) v.4.0.3 was used with default parameters. ClermonTyping (48) was used to classify isolates into phylogroups *in silico* with default settings.

### Phylogenetic analysis and genomic population

The *E. coli* K-12 MG1655 (GenBank: U00096.2) was chosen as the reference for the phylogenetic analysis, and core genome SNPs were called using the pipeline Snippy v.0.11.0 (https://github.com/tseemann/snippy). Subsequently, Gubbins v.2.4.1 (49) was employed for recombination removal. IQ-TREE v.2.2.0.3 (50) was used to generate a maximum-likelihood tree with the GTR model, and 1,000 bootstrap replicates based on cleaned core gene SNPs. PopPUNK v.2.6.0 (51) was employed to cluster all 445 genomes using a minimum *k*-mer size of 13 and a maximum *k*-mer size of 29. MLST profiles were determined via mlst (https://github.com/tseemann/mlst) and grouped into a clonal complex following Achtman’s scheme from PubMLST (52). Genomes that cannot be clustered into any defined clonal complex were classified as singletons. *K*-mer files were created for each genome based on the short-read data, employing SKA v.1.0 (53) with default settings. Subsequently, genome-to-genome pairwise SNP distances were computed using the “ska distance” function, also with default parameters. Genetically close pairs of *E. coli* were identified based on an SNP distance threshold of 100 or fewer (54).

### Source predictions by discriminant analysis of principal components

We employed a DAPC model to trace the potential origins of all CTX-M-producing *E. coli*. To establish this model, we accessed genomic sequences of 1,218 CTX-M-producing *E. coli* isolates from the NCBI databases, alongside metadata documenting their source of collection, with the last access date being 23 May 2025. Together with our 445 collected isolates, all genomes were categorized into human, animal, food, and environment. The SNP matrix of 1,218 isolates was then used to construct the DAPC model by the Adegenet package implemented in R 4.2.1 as previously described (55), and the remaining were used to assess the fitness of the model. Finally, the genetic origins of genomes in this study were then predicted using the constructed DAPC model.

### Mobile genetic element identification

PLASMe v.1.1 (56) was used to predict plasmid contigs with default settings. Chromosomal contigs were validated by mapping them against the *E. coli* K-12 reference genome using blastn with a threshold of 70% coverage and identity. To establish the putative *bla*_CTX-M_-carrying plasmids, a total of 72,556 raw plasmids were downloaded from PLSDB (57) on 13 January 2025. AMRFinderPlus v.4.0.3 was used to predict the *bla*_CTX-M_ genes, resulting in a total of 4,369 *bla*_CTX-M_-carrying plasmids (Table S10).

All contigs were aligned with curated *bla*_CTX-M_-carrying plasmid database, transposon database (TnCentral) (58), and the integron database (Integrall) (59). Briefly, pairwise alignments for all contigs were performed using NUCmer v.3.1, and all contigs with coverage exceeding 60% were selected as putative *bla*_CTX-M_-carrying mobile genetic elements. The pairwise genetic distance between putative *bla*_CTX-M_-carrying plasmid sequences was calculated using Mash v.2.3 with default settings. The genetic distance was used for plasmid cluster detection using the Louvain algorithm according to previous work, which optimizes modularity through iterative expectation-maximization, implemented via the Python-Louvain v.0.14 module. For replicon typing, plasmidfinder v.2.1.1 (60) and MOB-typer from MOB-suite (61) were used with default settings. IS elements were identified using ISEScan v.1.7.2.3 (62) and blastn against the ISfinder database (63), applying a threshold of 80% coverage and identity. The co-occurrence of IS elements and *bla*_CTX-M_ genes was assessed by identifying sequences located within a 10 kb proximity on the same contig.

### Contribution of genomic elements in the cross-sectoral dissemination of *bla*_CTX-M_

Pairwise comparisons were conducted among all isolates from different One Health sectors. To highlight the sharing between human and non-human sources, pairwise genomes were categorized into two groups: human chain and non-human chain. The human chain consisted of pairwise genomes where one genome was from a human origin and the other was from a non-human origin. The non-human chain included pairs where both genomes were from non-human sectors. For each pairwise genome comparison, we identified the *bla*_CTX-M_ dissemination scenarios as follows: (i) clonal dissemination mode, characterized by an identical clonal complex, *bla*_CTX-M_ gene, and plasmid cluster; (ii) plasmid dissemination mode, characterized by an identical plasmid cluster and *bla*_CTX-M_ gene, but different clonal complexes; (iii) Tn/In dissemination mode, characterized by identical transposon or integron, *bla*_CTX-M_ gene, and different plasmid clusters; and (iv) no linkage, characterized by unrelated cases. The relative frequency of each dissemination mode was calculated for each pairwise cross-sector comparison.

### Statistical analysis and visualization

To analyze the proportions of CTX-M-producing *E. coli* exhibiting specific antimicrobial resistance profiles across One Health sectors, we employed the chi-square test. Statistical significance was determined using a *P* value with a threshold of less than 0.05. Ninety-five percent confidence intervals were calculated. The statistical significance among the length of plasmids across clusters was determined using the Kruskal-Wallis test. Plots were primarily generated in R using the ggplot2 package (64, 65), with additional network visualization using Cytoscape v.3.10.1 (66).

## Data Availability

The genetic data are available from NCBI BioProject under accession code PRJNA1275600. All codes used in this study are available at https://github.com/JasonJiang42/HK_One_Health_analysis.

## SUPPLEMENTAL MATERIAL

Fig. S1 spectrum.03551-25-s0001.tiff Comparison of SNP distances of paired genomes within and between clusters.

Fig. S2 spectrum.03551-25-s0002.tiff Phylogenetic tree of 445 CTX-M-producing *E. coli* isolates aligned with antibiotic resistance genes.

Fig. S3 spectrum.03551-25-s0003.tiff Phylogroups and CTX-M alleles of 445 CTX-M-producing *E. coli* isolates across One Health sectors.

Fig. S4 spectrum.03551-25-s0004.tiff Comparison of pairwise SNP distance across One Health sectors.

Fig. S5 spectrum.03551-25-s0005.tiff Distribution of blaCTX-M genes across One Health sectors.

Fig. S6 spectrum.03551-25-s0006.tiff Cross-sectoral sharing of *bla*_CTX-M_ and regional distribution of *bla*_CTX-M_ genes.

Fig. S7 spectrum.03551-25-s0007.tiff Adjacent IS distribution among genomes with undetermined Tn/In.

Supplemental tables spectrum.03551-25-s0008.xlsx Tables S1 to S10.

ASM does not own the copyrights to Supplemental Material that may be linked to, or accessed through, an article. The authors have granted ASM a non-exclusive, world-wide license to publish the Supplemental Material files. Please contact the corresponding author directly for reuse.
