# Supplementary figures and images for: Cross-sectoral sharing of CTX-M-producing Escherichia coli: a One Health analysis to understand dissemination modes
Source: Microbiol Spectr. 2026 Feb 19;14(4):e03551-25. doi: 10.1128/spectrum.03551-25 (PMC13055372; doi:10.1128/spectrum.03551-25)

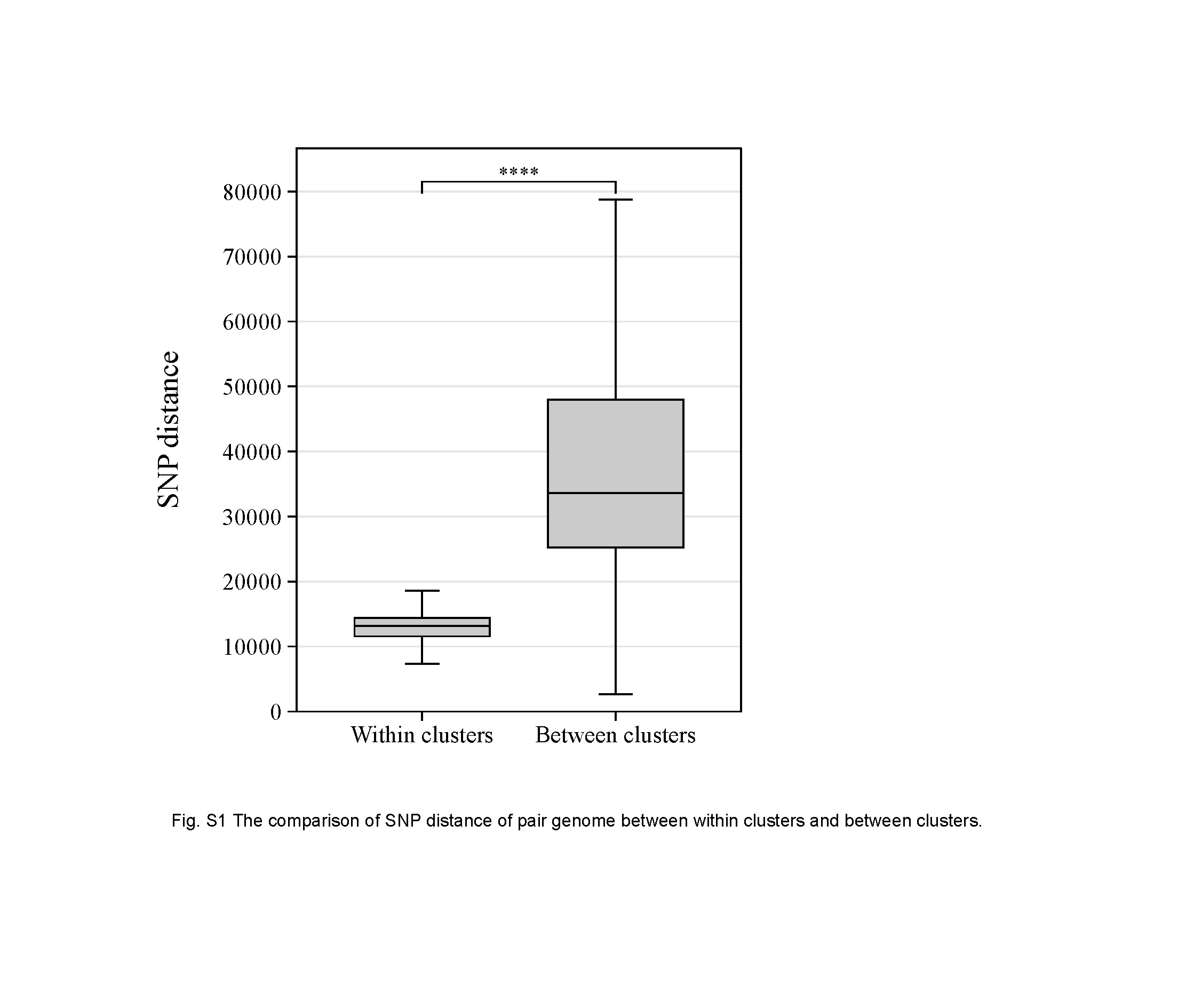

Supplement: Fig. S1 — Comparison of SNP distances of paired genomes within and between clusters. [file spectrum.03551-25-s0001.tiff]

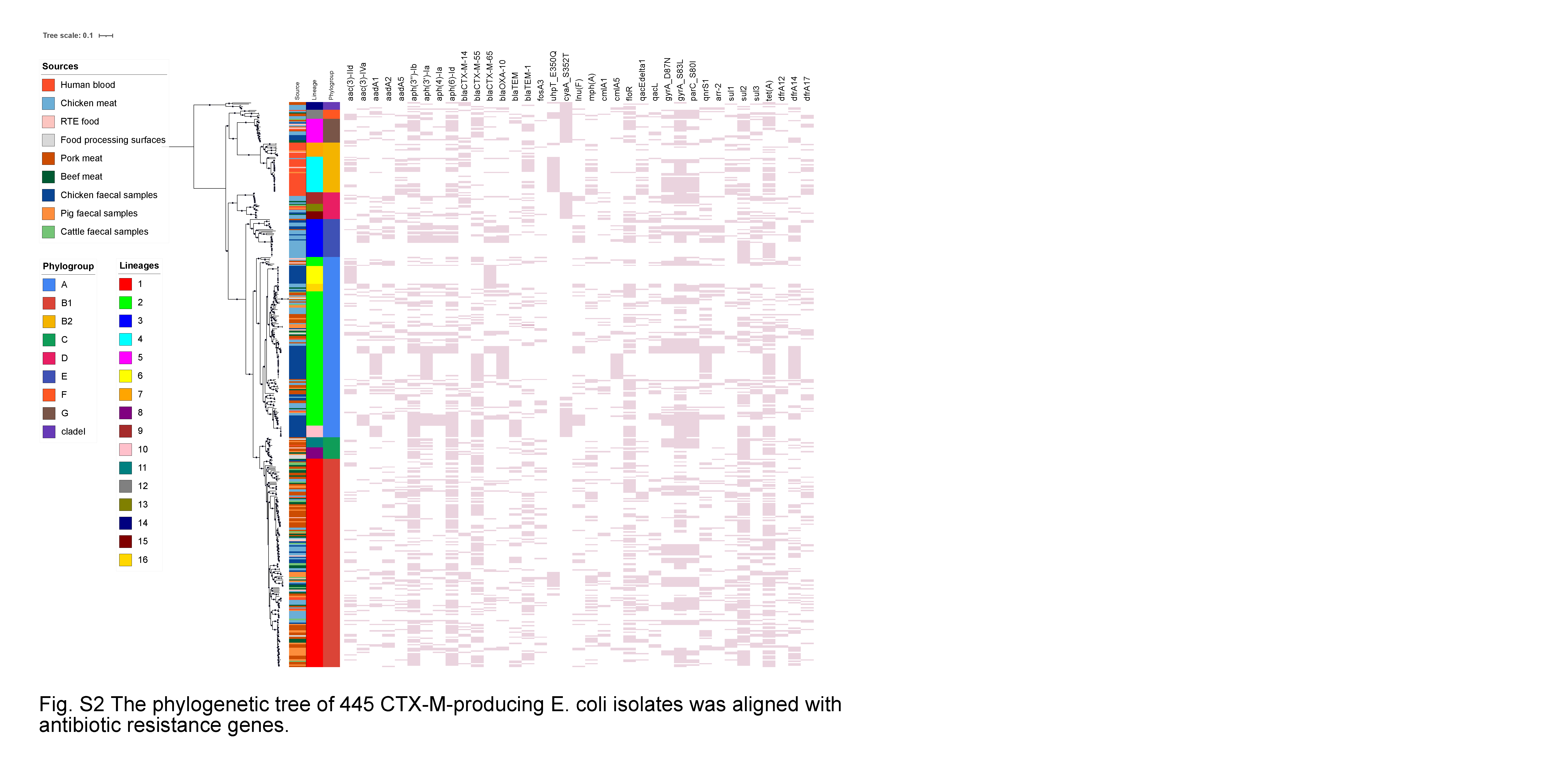

Supplement: Fig. S2 — Phylogenetic tree of 445 CTX-M-producing E. coli isolates aligned with antibiotic resistance genes. [file spectrum.03551-25-s0002.tiff]

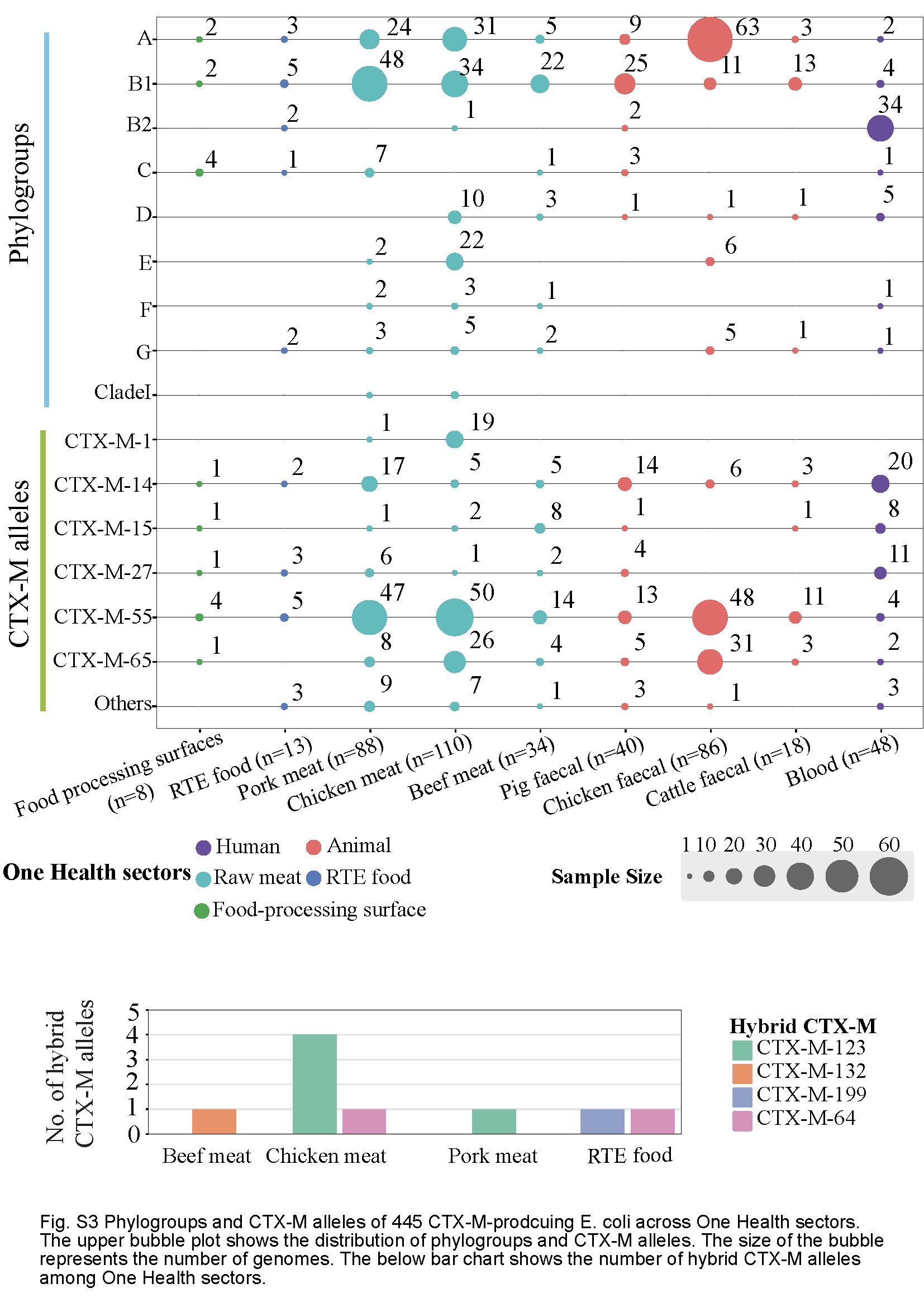

Supplement: Fig. S3 — Phylogroups and CTX-M alleles of 445 CTX-M-producing E. coli isolates across One Health sectors. [file spectrum.03551-25-s0003.tiff]

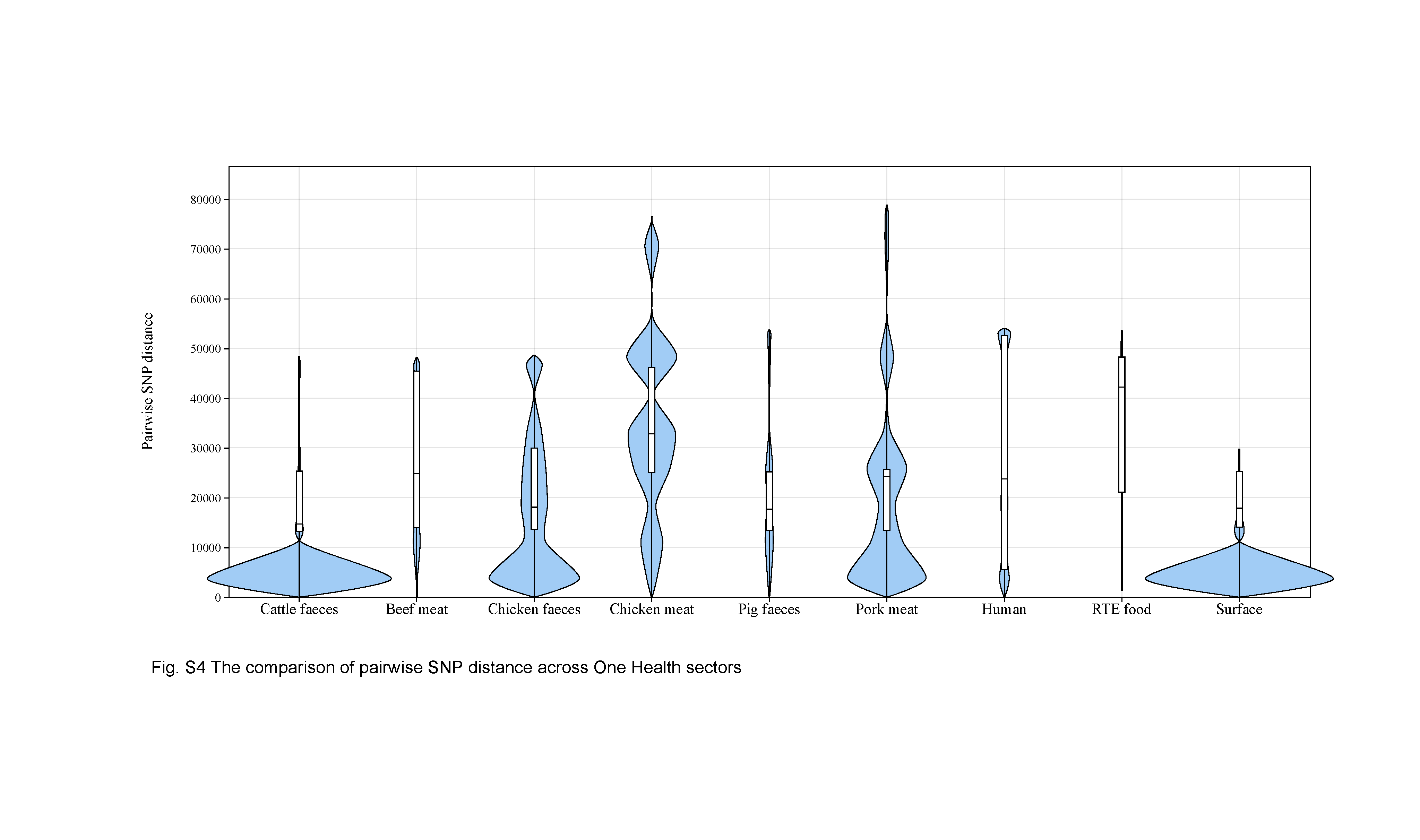

Supplement: Fig. S4 — Comparison of pairwise SNP distance across One Health sectors. [file spectrum.03551-25-s0004.tiff]

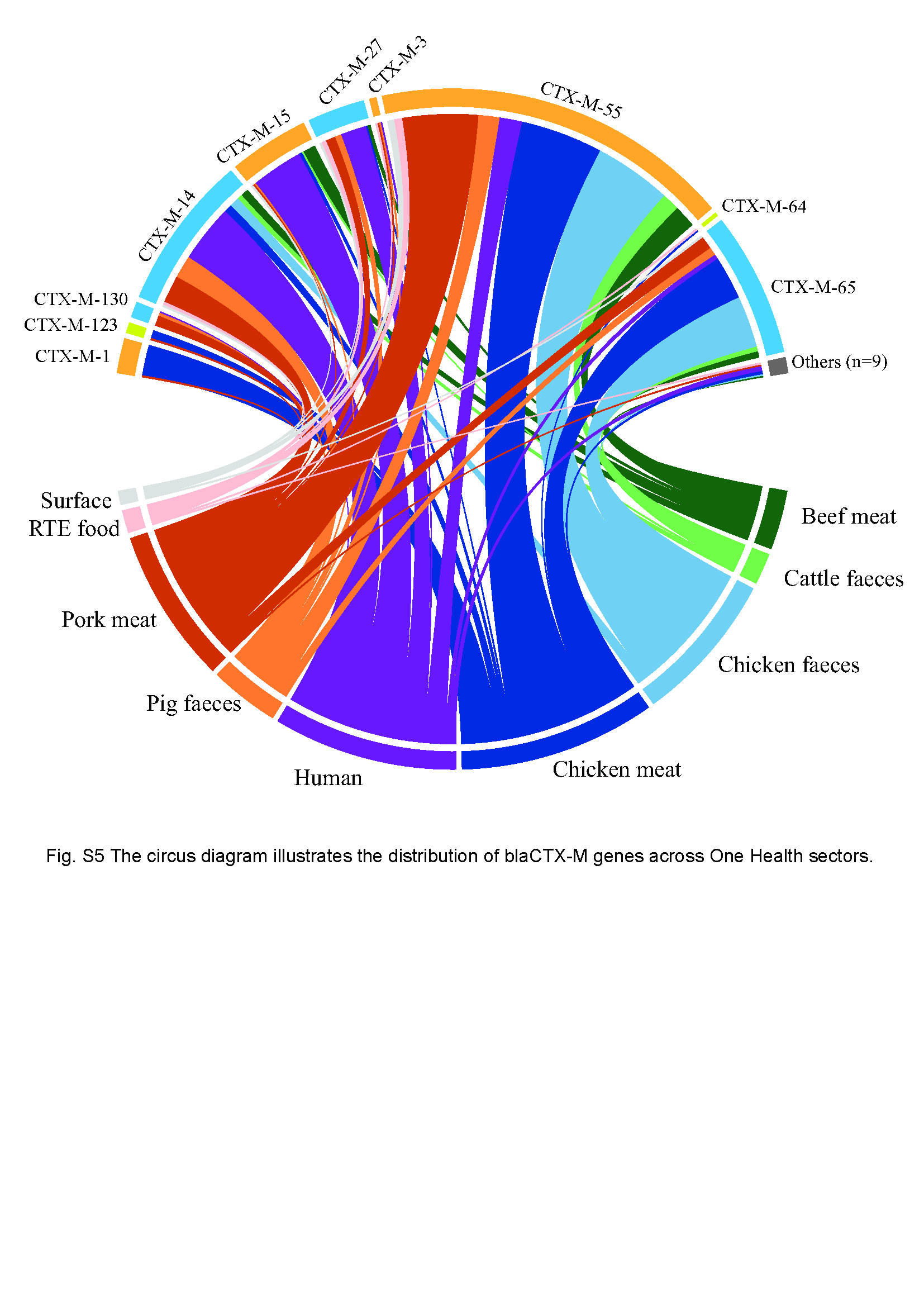

Supplement: Fig. S5 — Distribution of blaCTX-M genes across One Health sectors. [file spectrum.03551-25-s0005.tiff]

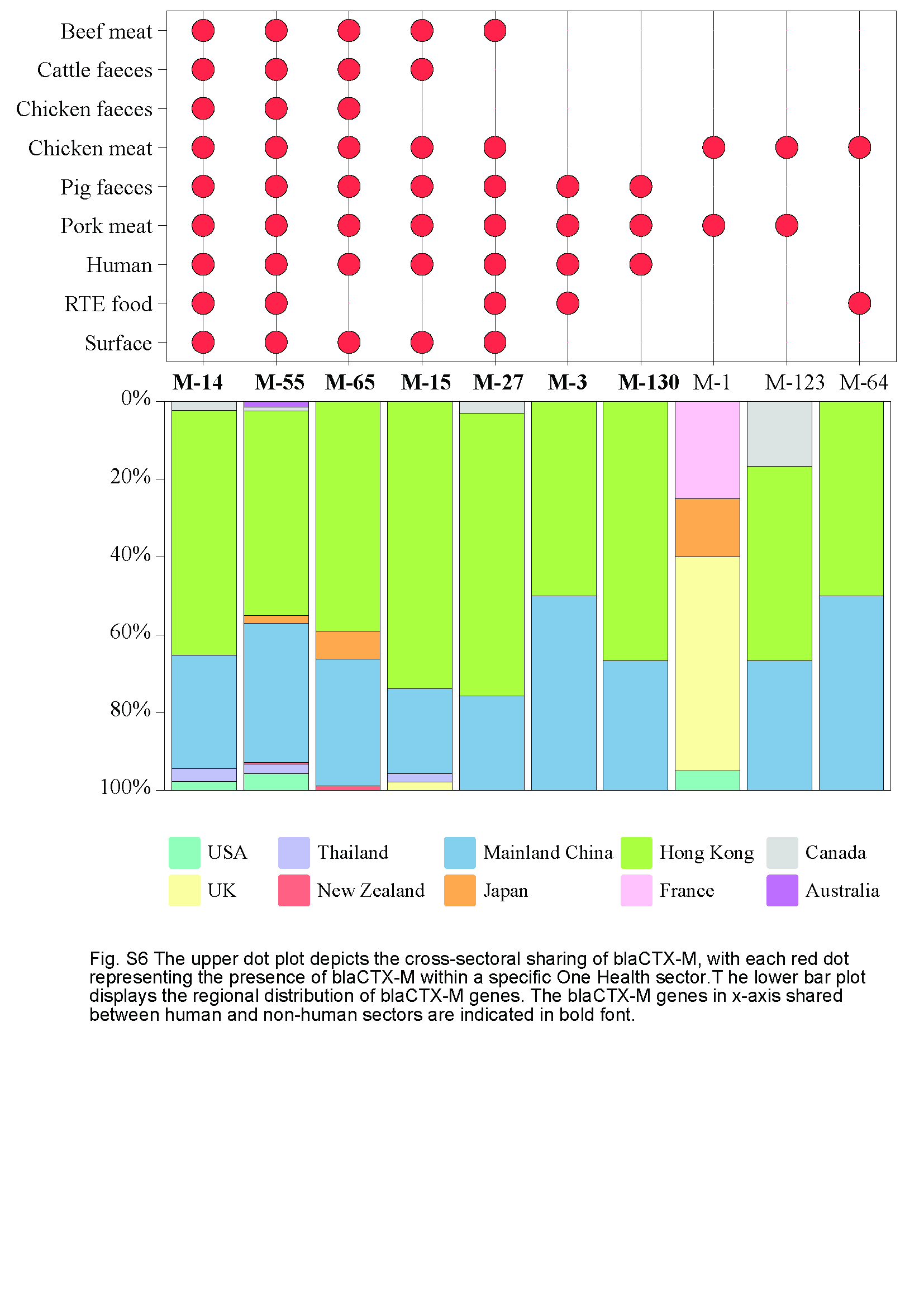

Supplement: Fig. S6 — Cross-sectoral sharing of blaCTX-M and regional distribution of blaCTX-M genes. [file spectrum.03551-25-s0006.tiff]

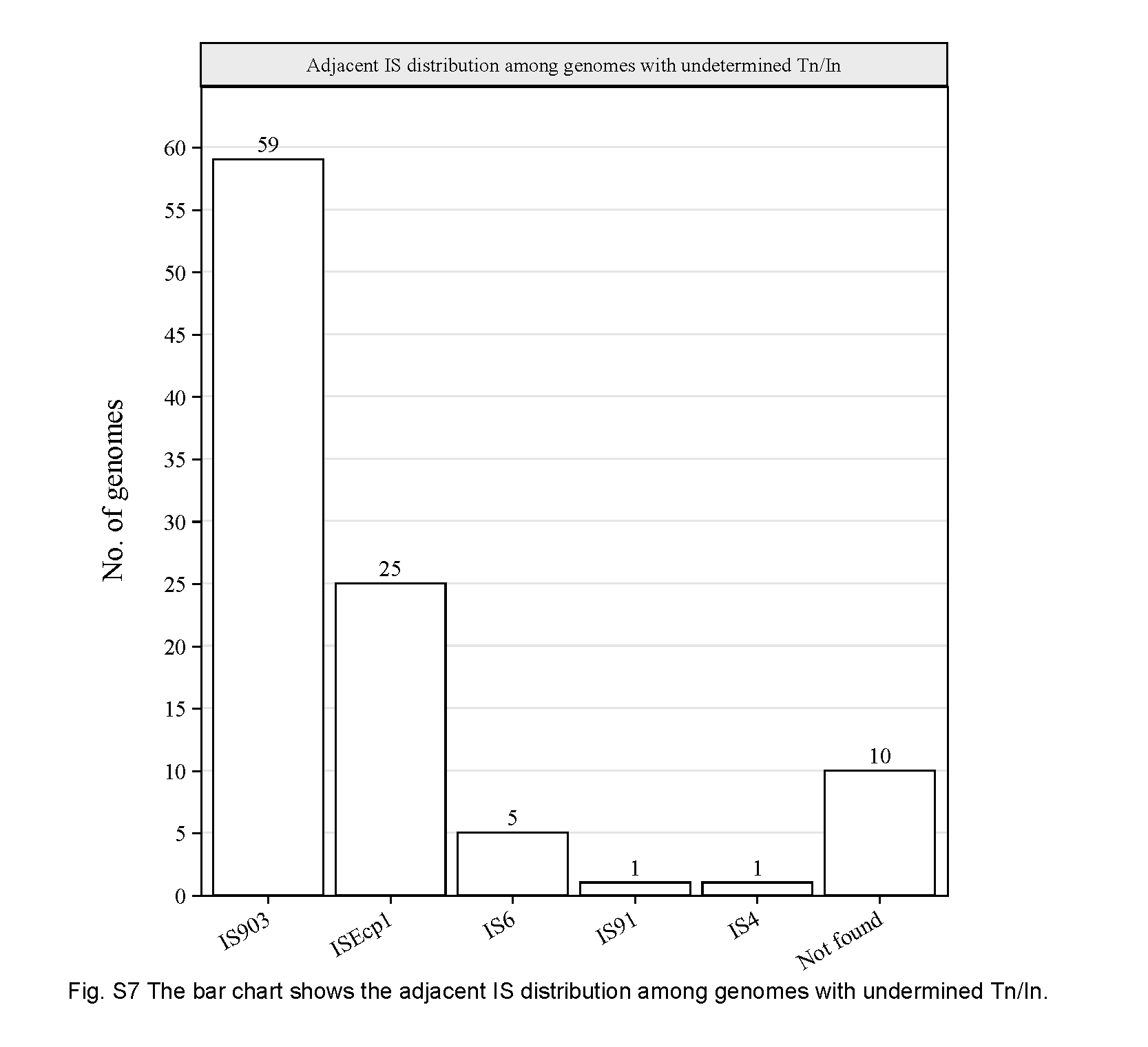

Supplement: Fig. S7 — Adjacent IS distribution among genomes with undetermined Tn/In. [file spectrum.03551-25-s0007.tiff]
